# Courtship Is a Major Factor of Mating‐Shortened Male Lifespan in an Egg Parasitoid

**DOI:** 10.1002/ece3.71932

**Published:** 2025-08-06

**Authors:** Zi‐Yin Wang, Jing Li, Jia‐Min Tang, Lan‐Feng Qiu, Hao‐Yuan Hu, Peng‐Cheng Liu

**Affiliations:** ^1^ The School of Ecology and Environment Anhui Normal University Wuhu Anhui China; ^2^ School of Laboratory Medicine Wannan Medical College Wuhu Anhui China; ^3^ Beijing Key Laboratory of Ecological Function Assessment and Regulation Technology of Green Space Beijing Academy of Forestry and Landscape Architecture Beijing China

**Keywords:** lipid metabolism, mating cost, parasitoid wasp, transcriptomic analysis

## Abstract

Mating is essential for population reproduction and survival, but individuals often incur costs of mating, including energy consumption and increased risks of death, predation, or disease transmission. Many studies on insects have reported a decreased post‐mating lifespan, and studies on the effects of mating on longevity have focused mainly on females. However, the effect of mating on lifespan has also been observed in males, and this process is thought to be more complex and distinctive. Sperm production and sperm transfer during copulation are believed to be the main factors involved in the mating‐induced reduction in male longevity due to significant energy investment, and this theory has been widely demonstrated in many insect species. Here, we found that mating has a negative effect on the longevity of males in an egg parasitoid species, *Anastatus disparis* . However, our results suggest that courtship rather than ejaculation is the main factor responsible for the reduced longevity of mated *A. disparis* males. Integrated transcriptomic analyses revealed the lots of upregulated genes and enriched Gene Ontology (GO) terms involved in lipid metabolism during male courtship. This finding suggested that lipid metabolism may be an important source of high energy expenditure during courtship. Besides, we discussed the role of increased lipid consumption for satisfying energy expenditure for courtship in the shortened lifespan of *A. disparis* males. In summary, our study provides comprehensive insights into post‐mating changes in male longevity and provides a basis for future mechanistic studies.

## Graphical abstract

Mating is vital for population survival but often leads to reduced post‐mating longevity in both sexes. In many insects, sperm‐related factors are thought to cut male longevity, while in *Anastatus disparis* , mating also affects male longevity, with courtship being the main cause. Transcriptomic analyses of *A. disparis* males showed lipid‐metabolism‐related changes that impact courtship energy costs and post‐courtship longevity. This study offers insights into post‐mating male longevity and a basis for future research.

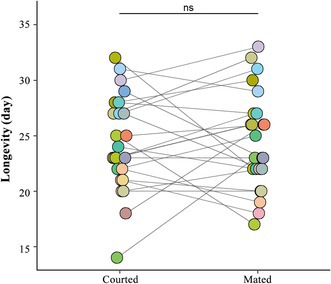

## 1 Introduction

For most species, including insects, mating is necessary for population reproduction, survival, and evolution (Miyatake 1997; Andersson and Simmons 2006). Typically, mating can offer fitness benefits, including both direct benefits (e.g., increased fecundity, acquired nutrients) and indirect benefits (e.g., increased longevity, selection of better quality offspring) (Arnqvist and Nilsson 2000; Chapman et al. 1998). However, mating is also a costly activity, particularly for females, which usually includes energy consumption and increased risks of death, predation, or disease transmission (Paukku and Kotiaho 2005). Many studies in insects, including *Aphelinus asychis* (Wang et al. 2021), the fruit fly (Partridge and Farquhar 1981) and *Trichogramma minutum* (Li et al. 1993), have reported a decreased post‐mating lifespan in females. Numerous studies have extensively investigated a series of factors that contribute to decreased female longevity after mating: energy consumption during mating (Kotiaho 2000; Wedell et al. 2002; Hunt et al. 2004; Billings et al. 2019), reduction of resource allocation to maintain survival (Browne 1993; Michaud 1994; Wheeler 1996; Pérez‐Maluf and Kaiser 1998), transfer of toxic compounds with spermatozoa during copulation (Das et al. 1980; Davey 1985; Chapman et al. 1995; Gems and Riddle 1996; Neubaum and Wolfner 1998), and increased vulnerability to predation, sexual diseases, parasites, and pathogens (Arnqvist and Nilsson 2000; Morrow et al. 2003). Generally, the costs must be balanced against the fitness benefits of mating (Andersson 1994; Johnstone and Keller 2000; Blanckenhorn et al. 2002).

However, the effect of mating on lifespan has also been observed in males (Kotiaho and Simmons 2003; Koliada et al. 2020; Partridge and Farquhar 1981; Metzler et al. 2016). Sperm and fluid production and transfer during copulation are considered the main factors responsible for the reduction in male longevity caused by mating because of the significant energy investment; this relationship has been repeatedly confirmed in multiple insect species (Prowse and Partridge 1997; Tregenza and Wedell 2002; Byrne and Rice 2006; Papadopoulos et al. 2010). For example, studies on the seed beetle *Callosobruchus maculatus* and the bed bug *Cimex lectularius* revealed that a significant energy investment in the transfer of sperm during copulation results in a reduction in male longevity (Crudgington and Siva‐Jothy 2000; Stutt and Siva‐Jothy 2001; Paukku and Kotiaho 2005; Brown et al. 2009). However, a study in *Drosophila melanogaster* revealed that germline ablation in males caused a slowing down in mortality late in life and an extension of the maximum (but not necessarily median) lifespan (Barnes et al. 2006). Furthermore, in addition to copulation, courtship is another component of male mating behaviour (Partridge and Farquhar 1981; Clutton‐Brock and Langley 1997; Savalli and Fox 1998; Kotiaho 2001; Kotiaho and Simmons 2003) that is typically mediated by pheromones. Research on *D. melanogaster* has shown that the perception of female sexual pheromones through a specific gustatory receptor expressed in a subset of foreleg neurons in males rapidly and reversibly decreases fat stores, reduces resistance to starvation, and limits the lifespan together with neurons that express the reward mediating neuropeptide F (Gendron et al. 2014). Accordingly, the negative effects of copulation and pheromone perception on lifespan likely involve distinct molecular mechanisms (Shi et al. 2017). In summary, the process by which mating affects lifespan in males is thought to be more complex and distinctive (LeBas and Marshall 2000), but many studies have focused mainly on the model species *D. melanogaster* (Aigaki and Ohba 1984; Koliada et al. 2020; Bretman et al. 2013), and the underlying molecular mechanism has been less studied (e.g., Hoffman et al. 2021). However, it has not been well investigated in insects other than the model organism *D. melanogaster* and a few other species, which may limit the comprehensive understanding of the mating cost on male lifespan.

Parasitoids are insects that are well known for their ability to control pests biologically (Wang et al. 2019) and for their use in theoretical research studies (Godfray 1994; Hardy and Wajnberg 2023). The cost of mating on longevity has been studied in parasitoids, but research has focused mainly on females (Carpenter 1995; Jacob and Evans 2000; King 2002). The sex determination of Hymenopteran parasitoids is haplodiploid, in which males develop from unfertilised eggs and are haploid, whereas females develop from fertilised eggs and are diploid (Cook 1993; Heimpel and De Boer 2008). Thus, mating may be more necessary for males than for females because virgin females can produce offspring, although the sex of all these offspring is male, and the effect of mating on male longevity requires more research. Here, *Anastatus disparis* (Hymenoptera: Eupelmidae), an important egg parasitoid of several noxious forest pest species of Lepidoptera (e.g., *Lymantria dispar* and *Odonestis pruni*) (Yan et al. 1989; Li and Lou 1992), was used as an experimental model to explore the effects of mating on male lifespan. In the wild, female *A. disparis* can live for more than 30 days (1 month), whereas males live for only 1 week (Yan et al. 1989). The longevity of both females and males in the wild is significantly longer than that of individuals reared indoors and fed honey water daily. Successful mating behaviour involved a progressive series: male courting, female reception, ovipositor insertion, and post‐mating (e.g., male lying on the female back) (Liu et al. 2021). In this study, the effect of mating on the longevity of males was first evaluated in *A. disparis* . Consistent with several studies (e.g., Crudgington and Siva‐Jothy 2000; Stutt and Siva‐Jothy 2001; Paukku and Kotiaho 2005; Brown et al. 2009), there might be a significant energy investment in the transfer of sperm during copulation in *A. disparis* . Thus, we expected that mating may have a negative effect on the longevity of *A. disparis* males. Alternatively, it is possible that mating can cause an increase in longevity of *A. disparis* males or have no significant effect. Crucially, in either case, we expect to see a difference in longevity between the virgin males and mated males. Like in most insect species, successful mating behaviour in *A. disparis* males involves a progressive series of behaviours, including courtship and copulation. Accordingly, the effect of each major mating behaviour on male lifespan was considered, as well. Finally, transcriptomic profiling is thought to be a widely used approach to provide answers to many biological problems (Wang et al. 2009; Zhang et al. 2023; Rani and Sharma 2017). Accordingly, in addition to the cost of mating on males' longevity in phenotypes, differences in transcription after mating were also investigated in this study in order to provide comprehensive insights into the molecular mechanisms underlying the biological traits associated with mating that cause changes in male longevity.

## 2 Materials and Methods

### 2.1 Parasitoid and Host

An *A. disparis* colony was established from a population reared on *Lymantria dispar* egg masses collected in Tongliao city, China (43°62′ N, 122°25′ E), in December 2019 and subsequently maintained on *Antheraea pernyi* eggs. Each female was provided with 20–30 *A. pernyi* eggs for oviposition, after which the hosts were incubated at 26°C–28°C. Eggs of *A. pernyi* were obtained through laparotomy of adult female abdomens, and eggs were maintained at 0°C. In *A. disparis* , males engage in extreme fighting behaviour to secure mating opportunities (Liu et al. 2020; Wang et al. 2022), and this behaviour can directly lead to physical injuries that affect male longevity. Therefore, to avoid mating and fighting among adults before the start of the experiment, we isolated the parasitised hosts individually in polyethylene tubes (height: 7.5 cm; diameter: 1 cm). Approximately 18 days before adult eclosion, females and males were checked daily and grouped by eclosion date. Additionally, *A. disparis* is a typical quasi‐gregarious species whose hosts are spatially aggregated, but only one parasitoid adult is produced per host (Yan et al. 1989). The peak eclosion period was between 9:00 a.m. and 12:00 p.m., and all the experimental parasitoids were collected during this period at 1 day of age.

### 2.2 Effects of Mating Behaviours on Male Longevity

To explore whether male mating behaviour influences longevity, we selected newly emerged virgin (*n* = 42) and mated (*n* = 42) males (i.e., 1 day old); raised them in incubators at a temperature of 26°C ± 0.5°C, a relative humidity (RH) of 70% ± 5%, and a photoperiod of 14 L:10 D; and recorded their lifespan. Owing to the extreme fighting behaviours of *A disparis* males during mating (Liu and Hao 2019a), each male (virgin and mated) was isolated in cylindrical boxes (height: 5 cm, diameter: 10 cm) and received honey water daily (honey: water = 2:3 vol/vol). To obtain mated males, a newly emerged female (i.e., 1 day old) was supplied to a virgin male (i.e., 1 day old) for mating, and mating behaviour was observed in a cylindrical arena (height: 1 cm, diameter: 5 cm). Each male was inspected twice daily, at 10 a.m. and 10 p.m., and the date of death of each male was recorded.

### 2.3 Effects of Courtship and Ejaculation Mating Behaviours on Male Longevity

In *A disparis*, the major mating steps for males include courtship and copulation (Liu et al. 2021). Accordingly, the effect of each main event on longevity was considered. Because *A disparis* females are monogamous (only mate once in a lifetime), virgin males only display courtship behaviours (without ejaculation) to mated females (Liu and Hao 2019b). In general, most *A. disparis* males exhibited courting behaviours within 5 min, and mating behaviour concluded after the experiment began (Liu et al. 2021). Thus, to calculate the effect of courtship on male longevity, a 1‐day‐old virgin male was introduced into a cylindrical arena (height: 1 cm, diameter: 5 cm) containing a 1‐day‐old mated female for 5 min. We recorded video of the entire process (ONTOP, X2S; Dinghaojia, China) and excluded the males that did not display courtship behaviours from subsequent experiments and analyses. After 5 min, the male was moved to a cylindrical box (height: 5 cm, diameter: 10 cm) and fed honey water daily until death. A total of 75 repetitions were conducted, and virgin males without any exposure to females were included as controls (*N* = 42). Additionally, the effects of prolonged courtship on longevity were investigated because one male can mate multiple times in its lifetime (e.g., a newly eclosed male can mate with ~9 females in 3 h, Liu et al. 2021), and males still exhibit fanning and courting behaviours toward mated females. Thus, the lifetime courtship behaviours for a male may not be limited to the behaviours expressed in the 5 min experiments above; therefore, a 1‐day‐old virgin male was introduced into a cylindrical arena (height: 1 cm, diameter: 5 cm) containing a 1‐day‐old mated female for 10 min (*N* = 24), 20 min (*N* = 24) or 30 min (*N* = 23). After the experiment, all the tested males were fed honey water until death.

To determine the effect of ejaculation on male longevity, we compared the longevity of virgin males that only displayed courtship behaviours without ejaculation (i.e., in the presence of mated females) to that of mated males (i.e., complete mating including courtship and ejaculation). One 1‐day‐old virgin male was provided to a virgin female, and the duration from the introduction to ejaculation (courtship frequently occurred) was recorded via manual time‐keeping. Parasitoid adults (both females and males) were removed after mating, and the same mated female was subsequently introduced to another virgin male for a duration matching the time recorded in the previous step. Accordingly, by comparing the longevity of mated males to that of virgin males that only display courtship behaviours (successively introduced to the same female for the same duration), we calculated the effect of ejaculation on male longevity. A total of 24 groups of males were included. Each tested male was isolated in a cylindrical box (height: 5 cm, diameter: 10 cm) and fed honey water daily until death.

### 2.4 Differentially Expressed Genes (DEGs) During Male Courtship

The previous experiments showed that courtship has a negative effect on male longevity; therefore, the genes that were differentially expressed during male courtship were conducted by transcriptomic analysis. To acquire courting males, each 1‐day‐old virgin male was introduced into a cylindrical arena (height: 1 cm, diameter: 5 cm) containing a 1‐day‐old mated female for 5 min, and males that did not display courtship behaviours according to the video record were excluded. Virgin males (i.e., 1 day old) without any exposure to females were collected as the control group. Each group included three replicates, and each replicate included ~20 adult males. The whole bodies of adults in the same group were pooled in a plastic tube (1.5 mL) and snap frozen in liquid nitrogen for RNA extraction. Total RNA was extracted from whole parasitoids using TRIzol (Invitrogen), and 3 μg of total RNA was converted into cDNA using the TruSeq Stranded mRNA LT Sample Prep Kit (Illumina, San Diego) to construct cDNA libraries. To obtain raw reads, cDNA libraries were sequenced on an Illumina HiSeq X Ten platform, and 150‐bp paired‐end reads were generated. Then, reads containing adapters, poly‐N reads, and low‐quality reads were removed from the raw data by FASTX‐Toolkit (https://github.com/Debian/fastx‐ toolkit), yielding clean reads. All the clean reads were mapped to the *A. disparis* genome using HISAT2 (v.2.0.4). The fragments per kilobase of transcript per million mapped reads were calculated for the genes expressed using StringTie (v.2.0) (Florea et al. 2013). To obtain more information on gene expression, relaxed criteria for DEGs were defined as genes with at least 1.5‐fold expression changes and false discovery rate [FDR]‐adjusted *p* < 0.05, as determined via the R package DESeq2 (v.1.22.1). The GOseq R package was used to determine the significant enrichment of Gene Ontology (GO) terms in the DEGs, and an adjusted *Q* value < 0.05 was chosen as the significance cut‐off.

### 2.5 Data Analysis

All analyses were performed with R software (v.4.1.3). Survival analysis was performed to analyze the effects of mating behaviours (including courtship and ejaculation) on longevity using the Wilcoxon rank sum test. The Wilcoxon signed‐rank test was used to compare the longevity of mated males to the longevity of virgin males that only displayed courtship behaviours when introduced successively to the same female for the same duration. A generalised linear model (GLM) with a normal error structure and log transformation to normalise the residuals was used to analyze the differences among durations of courtship, ejaculation, and post‐mating in terms of mating behaviour and the effects of the duration of the experiment (e.g., 10, 20 and 30 min) on the duration of courtship. The effects of the duration of the experiment on the frequency of courtship were analysed by GLM using a Poisson distribution (quasi‐Poisson distribution for overdispersion) and a log link function.

## 3 Results

### 3.1 Effects of Mating on Male Longevity

**FIGURE 1:**
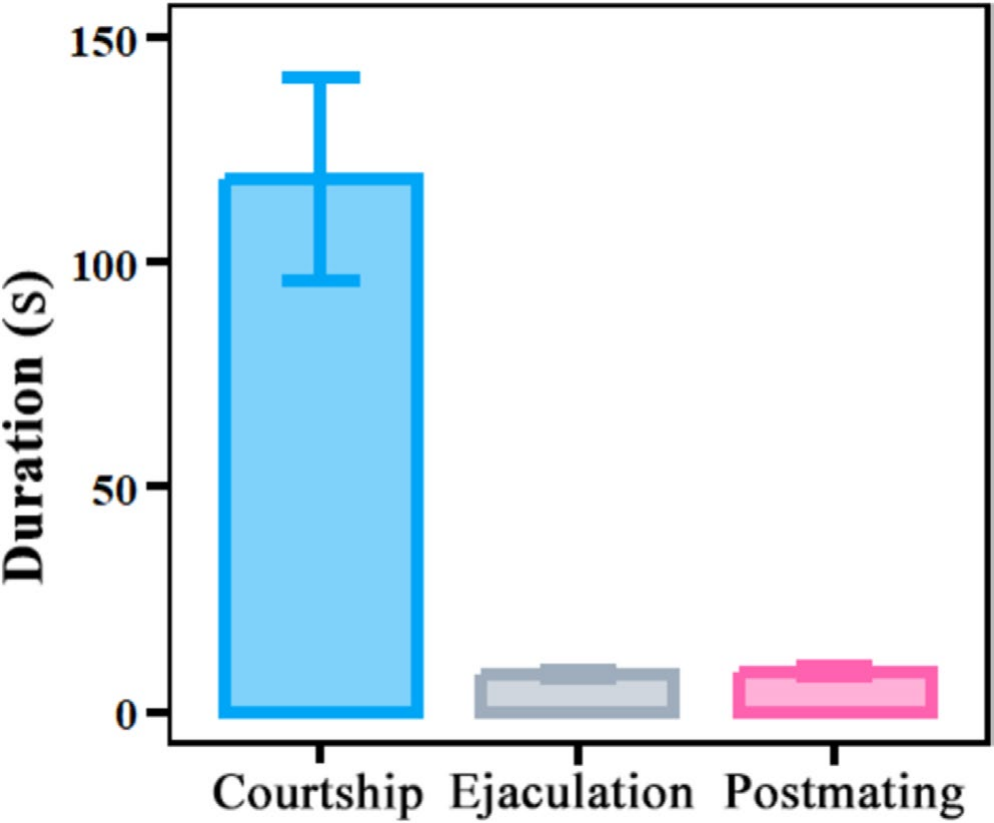
Durations of courtship, ejaculation and post‐mating in successful mating behaviour of male *Anastatus disparis* . The error bars indicate standard errors.

Successful mating behaviour of *A. disparis* males involves a progressive series of courtship, ejaculation, and post‐mating (e.g., males lying on the female's back) events. One male attempted courtship an average of 2.45 ± 0.34 times, which lasted 118.5 ± 22.43 s in total, before female reception. The duration of courtship was significantly longer than the durations of ejaculation (8.35 ± 0.82 s, *F* = 739.68, df = 82, *p* < 0.001) and post‐mating (8.9 ± 1.19 s, *F* = 352.98, df = 82, *p* < 0.001) (Figure 1).

**FIGURE 2:**
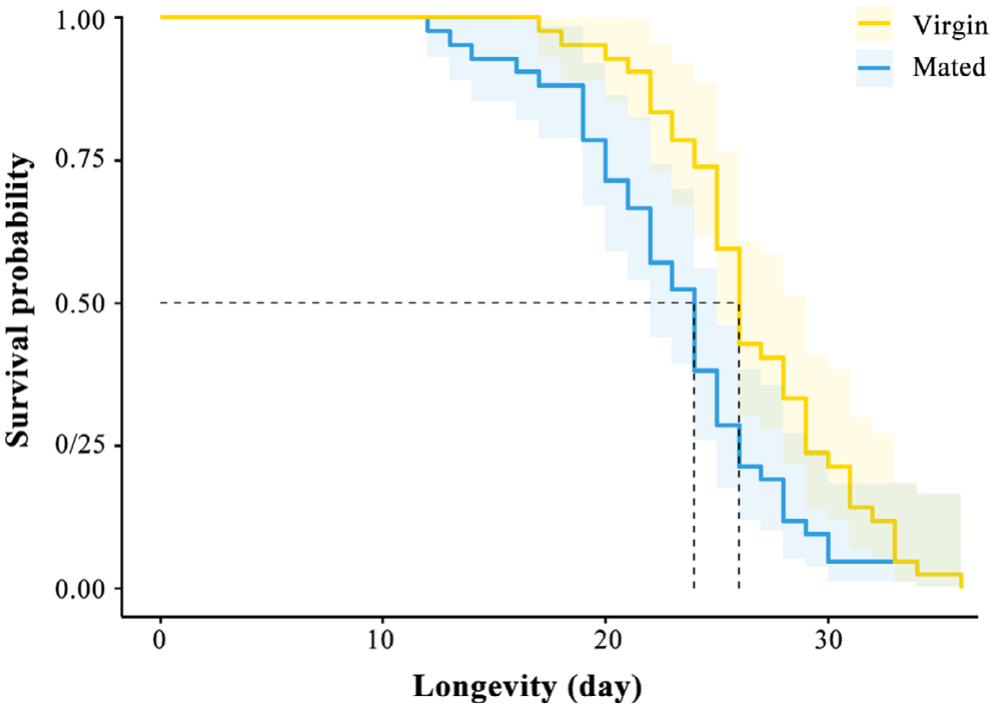
Survival curves of *Anastatus disparis* virgin males and mated males.

The mean survival time of virgin males without any exposure to females was 26.64 ± 0.66 days. The longevity of mated males was significantly shorter than that of virgin males (*χ* ^2^ = 10.578, df = 1, *p* = 0.001, Figure 2).

**FIGURE 3:**
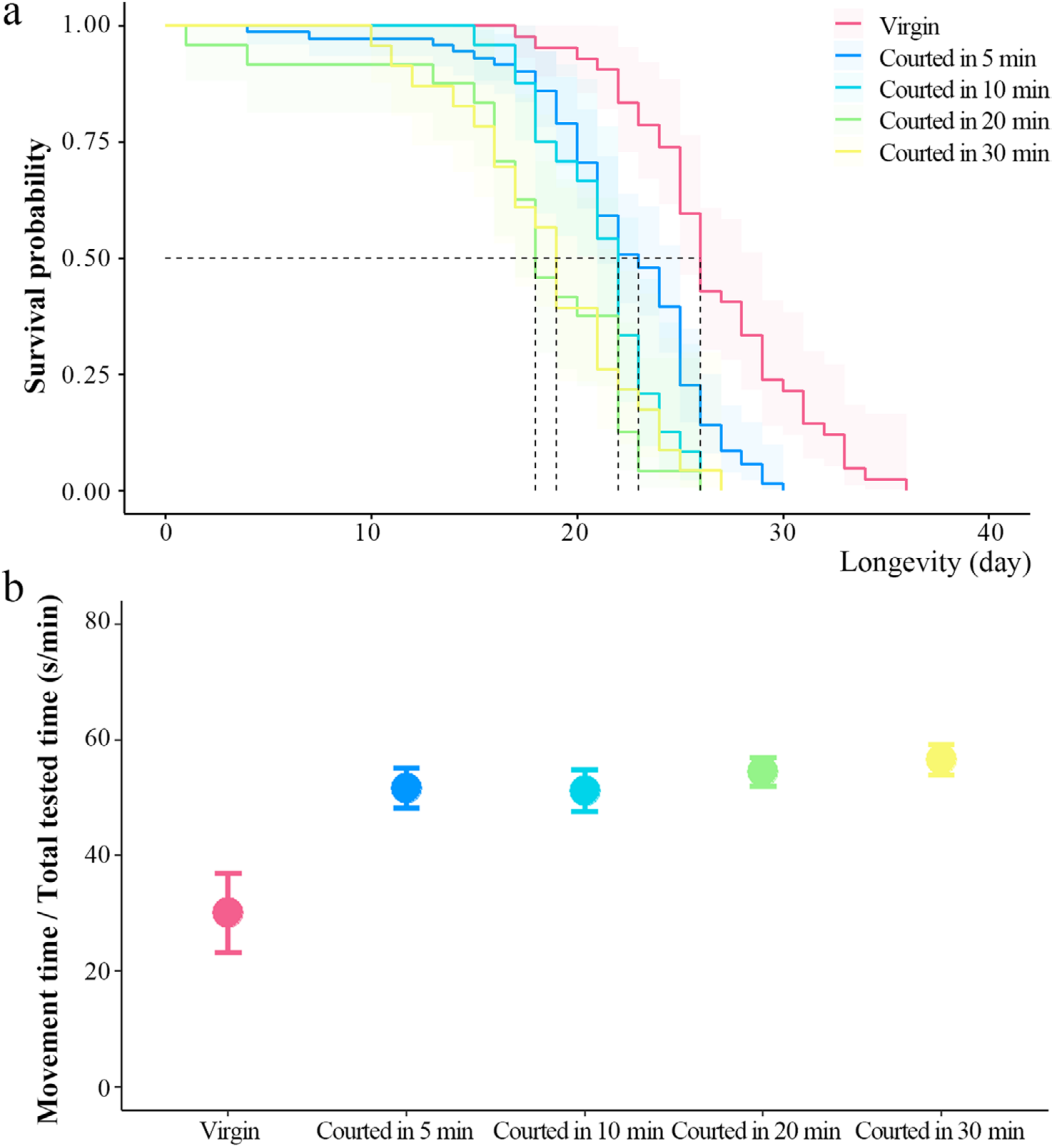
Survival curves (a) and movement duration (b) of virgin males and courted males over 5, 10, 20 and 30 min in *Anastatus disparis* . To acquire courted males, each 1‐day‐old virgin male was introduced into a cylindrical arena (height: 1 cm, diameter: 5 cm) containing a 1‐day‐old mated female. The error bars indicate standard errors.

**FIGURE 4:**
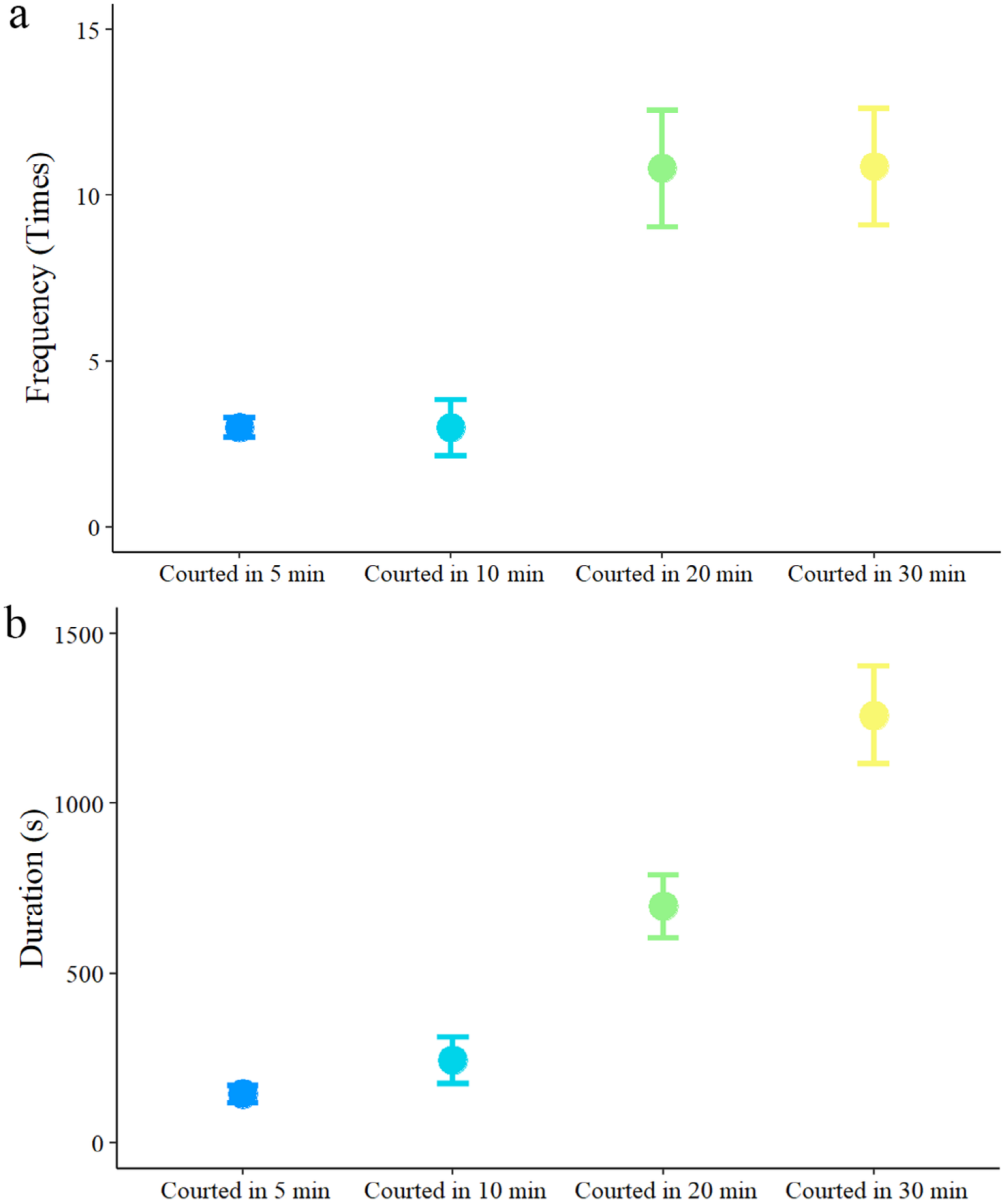
Courtship frequency and duration of courted males over 5, 10, 20 and 30 min in *Anastatus disparis* . To acquire courted males, each 1‐day‐old virgin male was introduced into a cylindrical arena (height: 1 cm, diameter: 5 cm) containing a 1‐day‐old mated female. The error bars indicate standard errors.

For males that only displayed courtship behaviours (i.e., introduced to a mated female) for 5 min, the male lifespan significantly decreased (*χ* ^2^ = 15.211, df = 1, *p* < 0.001, Figure 3a), and the courted male exhibited more active movement (*F* = 22.158, df = 115, *p* < 0.001, Figure 3b). In addition, when the experiment was extended to 10, 20, and 30 min for courtship, the courted male also exhibited more active movement (vs. 10 min, *F* = 18.432, df = 65, *p* < 0.001; vs. 20 min, *F* = 28.748, df = 65, *p* < 0.001; vs. 30 min, *F* = 33.649, df = 64, *p* < 0.001), and the frequency (*F* = 7.335, df = 3, *p* = 0.004, Figure 4a) and duration (*F* = 20.777, df = 3, *p* < 0.001, Figure 4b) of courtship significantly increased, but the longevity of males significantly decreased (*χ* ^2^ = 25.322, df = 4, *p* < 0.001, Figure 3a).

**FIGURE 5:**
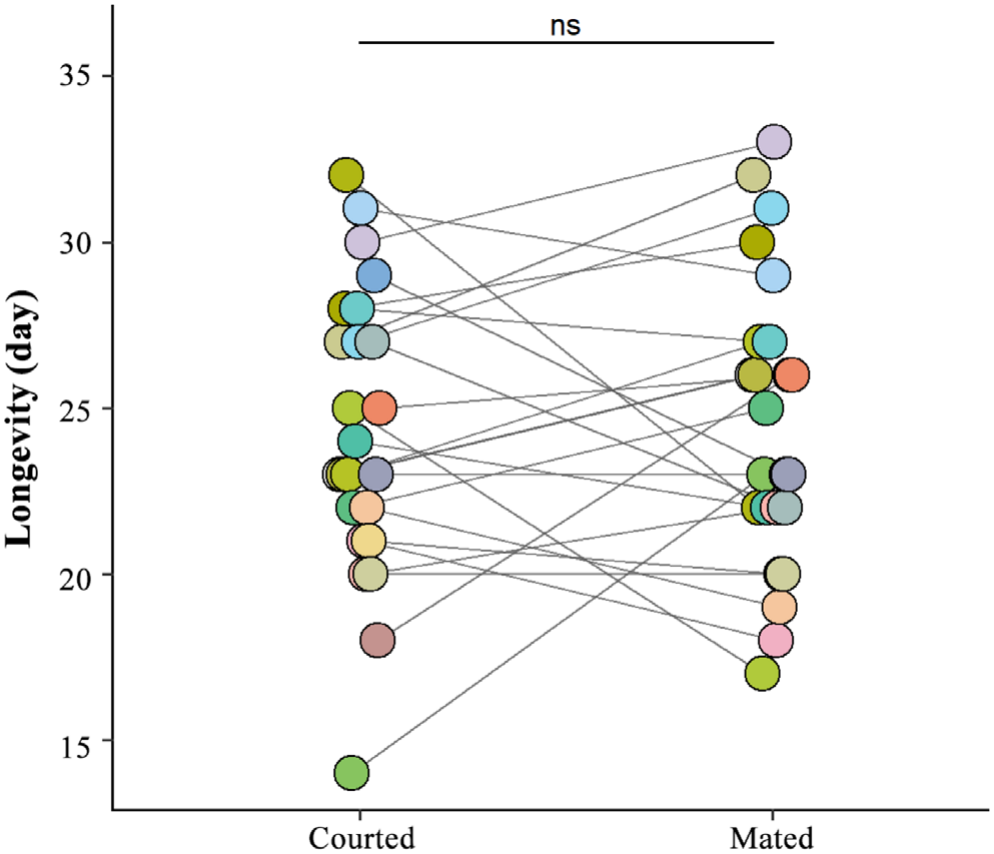
The longevities of mated males and virgin males that only display courtship when successively introduced to the same female for the same duration. n.s. means *p* > 0.05.

No significant differences in the longevity of mated males compared with that of virgin males that only displayed courtship behaviours when successively introduced to the same female for the same duration were detected (*Z* = −0.472, *p* = 0.648, Figure 5).

### 3.2 Transcriptomic Analyses in Courted Males

**FIGURE 6:**
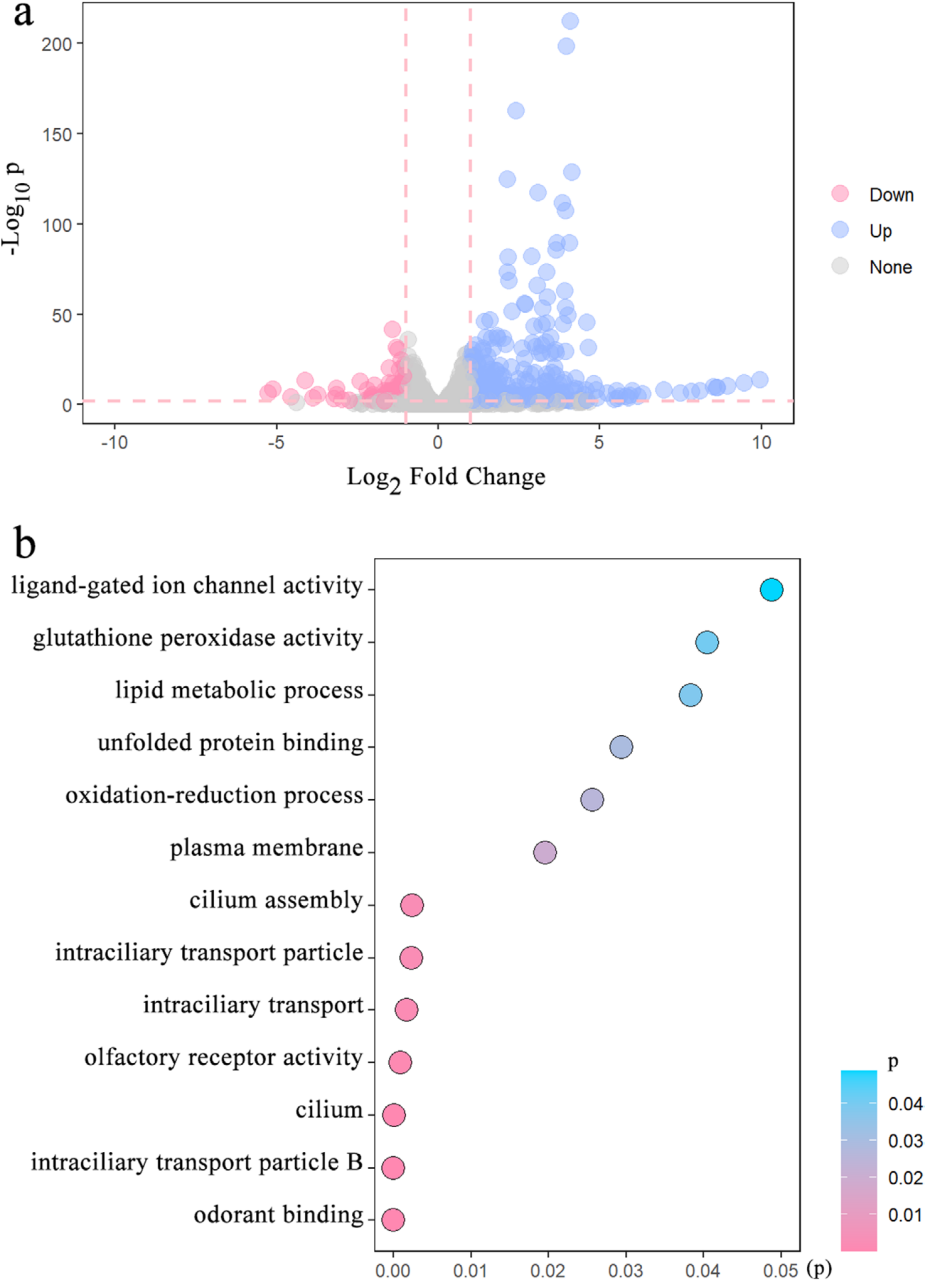
Transcriptomic analyses of courted males. (a) Volcano plot showing differential gene expression between courted males and virgin males. Compared with virgin males without any female exposure, 502 differentially expressed genes (DEGs) were identified in courted males. There were 384 significantly upregulated genes (shown in blue) and 118 significantly downregulated genes (shown in red). To acquire courted males, each 1‐day‐old virgin male was introduced into a cylindrical arena (height: 1 cm, diameter: 5 cm) containing a 1‐day‐old mated female for 5 mins. (b) Enriched GO terms of upregulated genes in courted *Anastatus disparis* males. The GOseq R package was used to determine the significant enrichment of GO subcategories, and an adjusted *Q* value < 0.05 was chosen as the significance cut‐off.

A total of 502 genes that were differentially expressed during male courtship were identified (Table S1). Of these, 384 genes had upregulated expression, and 118 genes had downregulated expression (Figure 6a). Among the upregulated genes, GO enrichment analyses revealed 13 enriched subcategories, including the molecular function terms odorant binding (GO:0005549), olfactory receptor activity (GO:0004984), unfolded protein binding (GO:0051082), glutathione peroxidase activity (GO:0004602) and ligand‐gated ion channel activity (GO:0015276); the cellular component terms intraciliary transport particle B (GO:0030992), cilium (GO:0005929), intraciliary transport particle (GO:0030990), and plasma membrane (GO:0005886); and the biological process terms intraciliary transport (GO:0042073), cilium assembly (GO:0060271), oxidation–reduction process (GO:0055114) and lipid metabolic process (GO:0006629) (Figure 6b). However, no enriched subcategories were identified for the downregulated genes.

## 4 Discussion

Consistent with many studies (Prowse and Partridge 1997; LeBas and Marshall 2000; Tregenza and Wedell 2002; Byrne and Rice 2006; Papadopoulos et al. 2010), in the egg parasitoid species *A. disparis* , mating has a negative effect on the longevity of males. The production of sperm and accessory fluids during ejaculation was considered the main factor that results in decreased longevity in many studies (Prowse and Partridge 1997; Tregenza and Wedell 2002). However, our results suggested that courtship rather than ejaculation is the main factor responsible for the decreased longevity of mated males.

It is now accepted that mating can carry fitness costs, which must be balanced against the fitness benefits of mating (Andersson 1994; Arnqvist and Nilsson 2000; Chapman et al. 1998; Johnstone and Keller 2000). The cost of mating for male *A. disparis* decreases longevity, which seems to be an acceptable cost, since males cannot reproduce on their own, and mating is the sole source of achieving a fitness benefit. Thus, males are generally expected to maximise their number of matings, and fitness increases with the number of matings (Andersson 1994; Bateman 1948). Many species can mate multiple times in a lifetime, including newly eclosed male *A. disparis* , which a can mate with approximately 9 females in 3 h (Liu et al. 2021). Multiple mating events come at increased cost, and several studies have shown a decreased longevity of males when the number of male mates increases (Wang et al. 2005; Oliver and Cordero 2009; Paukku and Kotiaho 2005). Our study revealed similar results in that the frequency and duration of courtship significantly increased, but the longevity of the males significantly decreased. In general, a greater investment in reproduction decreases survival in individuals; the traditional physiological explanation for this trade‐off is that survival and reproductive traits or processes compete for limited resources such as nutrients and energy (Cox et al. 2010). Numerous empirical studies have provided supporting evidence for this explanation (Bronson 1989; Gems et al. 1998; Tatar et al. 2001; Rion and Kawecki 2007; Judd et al. 2011). The behavioural data from *A. disparis* suggest that courtship consumes a substantial amount of energy, resulting in a decrease in male longevity. Successful mating behaviour in *A. disparis* usually involves a progressive series of male courting, copulatory organ insertion, and post‐mating; the duration of courtship in mating behaviour is > 6 times as long as the other behaviours combined, and the male becomes extraordinarily active during courtship. Besides, in our experiment design, virgin males only display courtship behaviours (without ejaculation) to mated females, but they occasionally attempted mounting. Thus, in addition to active motor behaviour, attempted mounting might also consume energy, which might be an additional potential cause of *A. disparis* mated male decreased longevity.

Transcriptomic analyses revealed enriched GO terms and the loss of upregulated genes involved in lipid metabolism (e.g., glucosylceramidase, lipase, and phospholipase A1, Table S1) in courting males. Considerable experimental evidence indicates close associations among reproduction, fat metabolism, and ageing (Thomsen and Hamburger 1955; Strong 1967; Socha et al. 1991; Wang et al. 2008; Goudeau et al. 2011; Lapierre et al. 2011; Judd et al. 2011; McCormick et al. 2012; Hansen et al. 2013). An increased life span is often negatively correlated with reproduction and positively correlated with increased fat storage, whereas fat metabolism influences the energetic cost of reproduction and may directly affect life span (Eijs and Van Alphen 1999; Colinet et al. 2007; Hansen et al. 2013). Accordingly, considerable energy may be consumed during courtship from lipid metabolism, resulting in a decrease in longevity in *A. disparis* males.

In addition, transcriptomic analyses revealed that GO terms (e.g., intraciliary transport particle B, cilium, intraciliary transport particle, intraciliary transport, cilium assembly) related to sperm motility were highly enriched for the upregulated genes. Cilia/flagella are present in the sperm tail and the underlying structure for sperm motility (Skinner 2018). In addition, the enriched GO subcategory “oxidation–reduction process” might suggest that this process is needed in males to provide an environment that supports sperm viability and motility (Wheeler 1996; Gillott and Friedel 1977). Accordingly, sperm motility seems to be activated during courtship, which may be in preparation for sperm to access the female genitals (e.g., seminal vesicles) after subsequent ejaculation. During courtship, in addition to the male's active motor behaviour, activated sperm motility is likely to be an important aspect of energy consumption and results in a decrease in longevity in *A. disparis* males.

The impact of fat consumption on longevity may be even more severe in parasitoids. Many studies have revealed that parasitoids usually lack adult lipogenesis and appear to be unable to convert excess carbohydrates to long‐term storage in the form of lipids; these studies have been performed in *Leptopilina heterotoma* (Eijs et al. 1998), *Macrocentrus grandii* (Olson et al. 2000), *Nasonia vitripennis* (Rivero and West 2002), *Eupelmus vuilletti* (Giron and Casas 2003), *Venturia canescens* (Casas et al. 2003), and *Diadegma insulare* (Lee et al. 2004). Consequently, several parasitoids are likely to be completely dependent on larval lipid resources in adulthood (Ellers 1996; Olson et al. 2000; Rivero and West 2002; Casas et al. 2003; Lee et al. 2004; Fadamiro et al. 2005; Kapranas et al. 2016; Snart et al. 2018). Thus, although *A. disparis* males feed on honey water after courtship, the high energy expenditure from lipid metabolism during courtship may play an important role in the shortened lifespan of males. The mechanism of lipogenesis in *A. disparis* adults should be identified in future studies. In addition, a study of *D. melanogaster* revealed that the perception of female pheromones decreased the longevity of males (Bretman et al. 2013; Gendron et al. 2014), and many genes encoding olfactory receptor proteins, which are believed to aid in the capture and transport of odorants and pheromones, were upregulated in courted males (including enriched GO terms) (Pelosi and Maida 1990). Thus, whether the perception of female pheromones during courtship decreases the longevity of *A. disparis* males should also be studied. In summary, our study integrated behavioural experiments, survival analyses, and transcriptomic analyses and revealed that courtship may be an important factor in mating‐induced decreased male longevity in *A. disparis* . This study provides comprehensive insights into post‐mating changes in male longevity and provides a basis for future mechanistic studies. Furthermore, our results also suggest that when studying the effects of mating behaviour on the lifespan or other phenotypes in other species, a more detailed consideration of the influence of each mating process may be necessary.

## Author Contributions

**Zi‐Yin Wang:** data curation (equal), investigation (equal), methodology (equal), software (equal), validation (equal), visualization (equal), writing – original draft (equal). **Jing Li:** data curation (equal), investigation (equal), methodology (equal), software (equal), validation (equal), visualization (equal), writing – original draft (equal). **Jia‐Min Tang:** formal analysis (equal), investigation (equal), methodology (equal), validation (equal). **Lan‐Feng Qiu:** data curation (equal), investigation (equal), resources (equal), software (equal), supervision (equal). **Hao‐Yuan Hu:** conceptualization (equal), data curation (equal), resources (equal), writing – review and editing (equal). **Peng‐Cheng Liu:** conceptualization (equal), data curation (equal), investigation (equal), resources (equal), supervision (equal), writing – review and editing (equal).

## Conflicts of Interest

The authors declare no conflicts of interest.

## Supporting information


**Table S1:** Differentially expressed genes (DEGs) during male courtship.

## Data Availability

The data that supports the findings of this study are available in the Supporting Information of this article.
